# Molecular Analysis of the Superior Efficacy of a Dual Epidermal Growth Factor Receptor (EGFR)-DNA-Targeting Combi-Molecule in Comparison with Its Putative Prodrugs 6-Mono-Alkylamino- and 6,6-Dialkylaminoquinazoline in a Human Osteosarcoma Xenograft Model

**DOI:** 10.3390/cells12060914

**Published:** 2023-03-16

**Authors:** Caterina Facchin, Ana B. Fraga-Timiraos, Julie Schmitt, Nadia Babaa, Naveet Pannu, Antonio Aliaga, Anne-Laure Larroque, Bertrand J. Jean-Claude

**Affiliations:** Cancer Drug Research Laboratory, The Research Institute of the McGill University Health Center (RI-MUHC), Department of Medicine, Faculty of Medicine and Health Sciences, McGill University, Montreal, QC H4A 3J1, Canada

**Keywords:** combi-molecule, osteosarcoma, DNA damage, EGFR

## Abstract

*Background*: ZR2002 is a dual EGFR-DNA-targeting combi-molecule that carries a chloroethyl group at the six-position of the quinazoline ring designed to alkylate DNA. Despite its good pharmacokinetics, ZR2002 is metabolized in vivo into dechlorinated metabolites, losing the DNA-alkylating function required to damage DNA. To increase the DNA damage activity in tumor cells in vivo, we compared ZR2002 with two of its 6-N,N-disubstituted analogs: “JS61”, with a nitrogen mustard function at the six-position of the quinazoline ring, and “JS84”, with an N-methyl group. *Methods*: Tumor xenografts were performed with the human Saos-2 osteosarcoma cell line expressing EGFR. Mice were treated with ZR2002, JS84 or JS61, and the tumor burden was measured with a caliper and CT/PET imaging. Drug metabolism was analyzed with LC-MS. EGFR and ɣ-H2AX phosphorylation were quantified via Western blot analysis and immunohistochemistry. *Results:* In vivo analysis showed that significant tumor growth inhibition was only achieved when ZR2002 was administered in its naked form. The metabolic dealkylation of JS61 and JS84 did not release sufficient concentrations of ZR2002 for the intratumoral inhibition of P-EGFR or enhanced levels of P-H2AX. *Conclusions*: The results in toto suggest that intratumoral concentrations of intact ZR2002 are correlated with the highest inhibition of P-EGFR and induction of DNA damage in vivo. ZR2002 may well represent a good drug candidate for the treatment of EGFR-expressing osteosarcoma.

## 1. Introduction

Over the past decades, we and others have demonstrated the feasibility of single molecules capable of blocking the epidermal growth factor receptor (EGFR) and inducing significant levels of DNA damage. The design of these molecules termed “combi-molecules” emerged from a principle designated by our group as “combi-targeting” [1,2,3,4,5,6,7,8] that seeks to combine two targeting functions within a single molecule with the purpose of inducing a tandem blockade of receptor-mediated DNA repair signaling while inducing strong levels of DNA damage in cells. We demonstrated that this strategy culminated into strong levels of apoptosis in tumor cells [9,10,11,12,13]. The studies on the designs of the combi-molecules led to their classification into two types: Type I combi-molecules [2,3,4,5,6,8,9,10,11,12] require hydrolysis to generate DNA-alkylating species, whereas type II combi-molecules [13,14,15,16,17,18] are chimeric systems capable of eliciting dual biological activity without a requirement for metabolic activation. In vitro, the most potent type II combi-molecules reported by our laboratory are mustard conjugates directly attached to the quinazoline ring [13,14,19]. Type I combi-molecules, because of their requirement for hydrolysis, present less favorable pharmacokinetics than type II; therefore, the further advancement of the principle in vivo was performed with type II combi-molecules carrying a 2-chloroethyl moiety on the quinazoline ring. We recently showed that one such molecule, named ZR2002 [17], exhibited strong antitumor potency in a brain tumor model [18]. However, despite its good pharmacokinetics, this molecule was found to be metabolized in vivo into a des-chloroethyl metabolite, termed RB10, losing the DNA damage activity and becoming a reversible and less potent EGFR inhibitor [15].

**Figure 1:**
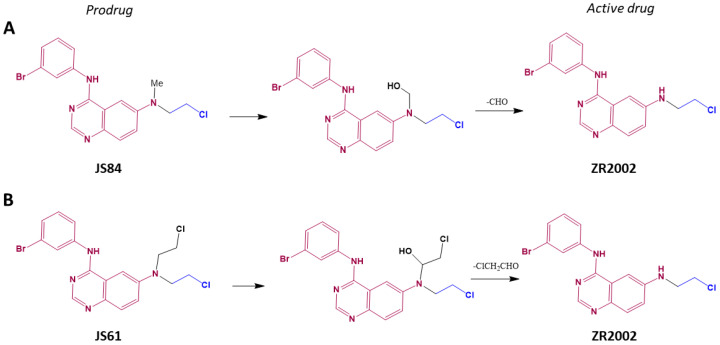
Proposed in vivo metabolism pathways toward conversion of the combi-molecules JS84 (**A**) and JS61 (**B**) to ZR2002.

In order to improve the potency of the dual-targeting cellular mechanism of ZR2002, we synthesized and previously compared in vitro the potency of its mono- with its 6-N,N-disubstituted derivatives [16]: JS61 (N,N-dialkylamino) and JS84 (N,N-monoalkylamino). In vitro, JS61 was capable of inducing high levels of DNA damage and moderate EGFR inhibitory potency. However, given that JS61 and JS84, by carrying at least one chloroethyl group, can be dealkylated through in vivo metabolism, we surmised that they could behave as stable prodrugs of ZR2002, increasing the concentration of the active combi-molecule ZR2002 in a tumor (Figure 1). Therefore, we predicted that despite their moderate in vitro activity, they might exhibit strong in vivo potency. To verify this hypothesis, we chose to study the effects of these drugs in vitro on a high-mortality tumor cell type, the Saos-2 osteosarcoma cell line expressing EGFR, and in vivo in a corresponding xenograft model. Since osteosarcoma is a disease in which 30–80% of cases express EGFR [20,21,22,23,24,25] and the standard chemotherapy involves cytotoxic DNA damaging agents [26], we believed that the Saos-2 model expressing EGFR [27] was the appropriate and relevant in vivo model to demonstrate the effects of targeted combi-molecules carrying a cytotoxic DNA-alkylating moiety. Nevertheless, we expanded the in vitro assays to additional osteosarcoma cells in a 2D monolayer culture to ascertain their growth inhibition profiles. While some studies on sarcoma have utilized 3D models for drug testing [28,29], the marked effect of liver metabolism on the activity of the molecules under study dictated the use of an in vivo model, which we chose to perform with the Saos-2 cell line. Herein, we challenge the hypothesis that the in vivo metabolism of 6-N,N-di-substituted derivatives into mono-substituted combi-molecules could enhance the potency of the dual EGFR-DNA-targeting mechanism by sustaining high concentrations of the dual-acting EGFR-DNA targeting species in tumor cells in vivo.

## 2. Materials and Methods

### 2.1. Cell Culture

Saos-2, U2-OS, MG-63, MNNG/HOS and KHOS/NP cells were purchased from ATCC. Saos-2 cells were cultured in RPMI 1640 (Wisent, Saint-Jean-Baptiste, QB, Canada) containing 10% FBS (Wisent) and supplemented with 12.5 mL of HEPES buffer (Wisent) and 500 µL of gentamycin (Wisent), 250 µL of amphotericin B (Wisent) and 170 µL of ciprofloxacin (Fluka Chemie GmbH, Buchs, Switzerland) as antibiotics. U2-OS cells were cultured in McCoy’s 5A (Wisent) containing 10% FBS and 1% of penicillin-streptomycin solution (Wisent). MG-63, MNNG/HOS and KHOS/NP were maintained in EMEM (Wisent) containing 10% FBS (Wisent) and 1% of penicillin-streptomycin solution (Wisent). All cell lines were maintained in a humidified incubator at 37 °C with 5% CO_2_.

### 2.2. Sulforhodamine B (SRB) Growth Inhibition Assay

To study the effects of the compounds on cell proliferation, cells were seeded with a volume of 100 µL at a density of 1000–3000 cells/well (i.e., 70% of confluence) in 96-well plates (Corning Inc., Corning, NY, USA). After 24 h of cell attachment on the culture plates in a humidified incubator at 37 °C with 5% CO_2_, they were treated with 100 µL of different drugs (with either ZR2002 or JS61 or JS84 [16] or gefitinib (Sigma, Saint Louis, MO, USA) or chlorambucil (Sigma, St. Louis, MO, USA), or their corresponding equimolar combination (chlorambucil + gefitinib), dissolved in media at different dose ranges and stored in the incubator for 5 days. Cells were then fixed with 50 µL of 50% trichloroacetic acid (Thermo Fisher Scientific, Waltham, MA, USA) for 2 h at 4 °C and subsequently rinsed with water and left to dry overnight. The cells were then stained with 100 µL of a 0.4% SRB solution (Alfa Aesar, Ward Hill, MA, USA) for 1.5 h at room temperature in a shaking device and, afterward, rinsed with 1% (*v*/*v*) acetic acid (Alfa Aesar, Ward Hill, MA, USA) 3 times and left to dry overnight. The dissolution of the colored complex was achieved using 200 mL of 10 mM Tris base (Thermo Fisher Scientific, Waltham, MA, USA), and the optical density was read with a Tecan Infinite 200Pro plate reader (Serial No. 1412003451) at a wavelength of 492 nm.

### 2.3. In Vivo Experiments

Animal experiments were executed in accordance with a protocol approved by the McGill Institutional Animal Care Committee (McGill University Health Centre Research Institute and McGill University, Montreal, Canada). Xenografts of the tumors were obtained via unilateral and subcutaneous injection (5 × 10^6^ cells in 100 µL of a PBS1x solution) into immunodeficient NSG male mice (N = 28; 8 weeks old; weight = 30 g; Charles River, Canada). The mice were maintained at optimal temperature (24 °C) and relative humidity (50%) on a 12/12-light/dark cycle with free access to water and food. The mice were monitored clinically every day; body weights were recorded and tumor volumes were measured with a caliper using the formula 4/3 π (length/2) (width/2)^2^ every other day. Once tumors reached 68 mm^3^, the mice were randomly divided into 4 groups: 1 was treated with ZR2002 (N = 7), another was treated with JS84 (N = 7), another was treated with JS61 (N = 7) and the last was treated with a vehicle (N = 7). The mice were treated every other day for 20 days, receiving a total of 10 doses via oral gavage of 200 µL of a ZR2002, JS61 or JS84 solution at a dose of 80 mg/kg body weight or the vehicle (control group). The drugs were dissolved in a solution of 25% of alcohol (Greenfield, Toronto, ON, Canada), 25% of Cremophor (Sigma-Aldrich, St. Louis, MO, USA) and 50% of 0.9% NaCl (Sigma-Aldrich, St. Louis, MO, USA). The vehicle solution used the same mixture without any drug.

In order to study the drug pharmacokinetics, 1 last dose (the 11th dose) was administered 3 h prior to sacrifice and after a break of 5 days from the previous drug administration.

### 2.4. Effects on 2′-Deoxy-2′-[^18^F] Fluoro-D-Glucose (^18^F-FDG) on In Vivo Cellular Metabolisms Using PET/CT Imaging

All PET/CT experiments were performed using nanoScan PET/CT for small animals (Mediso Medical Imaging Systems, Budapest, Hungary). The radiotracer 2′-deoxy-2′-[^18^F]fluoro-D-glucose (^18^F-FDG) was inoculated via 1 bolus injection in the tail vein (5.3 MBq in 100 µL). All animals were starved from the night before the experiment. The mice were kept awake for 45 min before the scans and weighed, and glycemia was measured in blood drawn from the caudal artery. Afterward, each mouse was placed in the scanner, with the tutor at the center of the field of view. Each PET/CT session consisted of a 30 min PET emission scan followed by a 10 min CT transmission scan. The PET/CT scans were conducted under anesthesia (isoflurane 2%; medical air (0.6 L/min)) delivered via a nose cone. Temperature and heart rate were monitored throughout the procedure using the Mediso system. Images were reconstructed using expectation maximization (EM) and ordered subset expectation maximization (OSEM), normalized, and corrected for scatter, dead time and decay. The CT volume-of-interest (VOI) was drawn manually on the CT and PET co-registered images. ^18^F-FDG accumulation was quantified as the mean standardized uptake value (SUV) between 20 and 30 min post-injection in a 3D volume-of-interest (VOI), which was named the metabolic volume (MV), delineated semi-automatically with iso-contours at a 40% threshold of the maximal SUV [30] in the tumor on PET/CT fusion slices using the PMOD software package v3.6 (PMOD Technologies Ltd., Zürich, Switzerland). In addition, another tumor volume-based parameter, called total lesion glycolysis (TLG), was calculated according to the product between the MV and the mean SUV. Furthermore, at the end of the imaging sessions, the tumors were resected and processed for ex vivo analysis.

### 2.5. Ex Vivo Analysis

At the end of the study, the mice were sacrificed, and the plasma and tumors were collected. The plasma was stored at −80 °C, and the tumor tissues were transversely cut into 2 parts: 1 was immediately frozen in liquid nitrogen and stored at −80 °C for Western blots and LC-MS analysis; the other was fixed in 4% paraformaldehyde and used for histology.

#### 2.5.1. Immunostaining

The tumors were fixed for 36 h in 4% paraformaldehyde, transferred to 70% EtOH and paraffin-embedded. Sections 4 µm thick were deparaffinized, rehydrated and incubated for 20 min at 37 °C with anti-P-EGFR (1–100, ab40815, Abcam, Cambridge, UK) or anti-P-H2AX (1–100, sc-517348, Santa Cruz Biotechnology, Dallas, TX, USA), followed by secondary Ab incubation with OmniMap anti-Rb HRP (760–4311, Roche, Basel, Switzerland) at room temperature for 16 min, followed by the detection kit ChromoMap Kit (760–4304, Roche, Basel, Switzerland), and the slides were then counterstained with hematoxylin, dehydrated, cleared and cover-slipped. The slides were digitally scanned using an Aperio scanner scope XT.

#### 2.5.2. Western Blots

Tumor cells were seeded in 6-well plates (Corning Inc., Corning, NY, USA) at 500,000 cells/well with a volume of 2 mL and allowed to attach in a humidified incubator at 37 °C with 5% CO_2_ overnight. The cells were scraped, harvested and lysed for 30 min over ice in RIPA 1× buffer with an anti-protease and anti-phosphatase inhibitor cocktail (Roche, Basel, Switzerland). The samples were centrifuged at 13,000 rpm for 20 min at 4 °C, and the supernatants were collected. All cell lysates had their proteins quantified, and equal quantities of proteins were prepared in Laemmli buffer (Sigma-Aldrich, St. Louis, MO, USA).

The tumor tissue samples were smashed with liquid nitrogen into a mortar, and the tumor powders were transferred to cold RIPA 1x buffer with an anti-protease and anti-phosphatase inhibitor cocktail (Roche, Basel, Switzerland) in ice for 1 h and sonicated. After centrifugation, the proteins were quantified and mixed in Laemmli buffer (Sigma-Aldrich, St. Louis, MO, USA), maintaining the same concentration.

Cell lysates or tissue lysates were loaded onto 4–20% SDS-PAGE gels (mini-protean TGx stain-free, Bio-Rad, Hercules, CA, USA) and transferred to PVDF membranes that were pre-wetted with methanol. The membranes were blocked with 5% BSA in PBS 1x with 0.1% Tween-20 and immunoblotted overnight with the following antibodies—P-EGFR (Tyr1068) (D7A5) (1:1000; #3777; Cell Signaling, Danvers, MA, USA), EGFR (1:1000; #2646; Cell Signaling, Danvers, MA, USA) and phospho-histone H2A.X (Ser139) (1:1000; #2577; Cell Signaling, Danvers, MA, USA)—for tumor-tissue-derived blots and immunoblotted with the EGFR antibody for tumor-cell-derived blots. The (HRP)-conjugated anti-rabbit secondary antibody (1:5000, sc-2004, Santa Cruz Biotechnology, Dallas, TX, USA) was used as a secondary antibody. Chemiluminescence detection was performed using an ECL kit (SuperSignal™ West Pico PLUS, Thermo Fisher Scientific, Waltham, MA, USA). The quantification of the immunoblots was performed on digitalized images using ImageLab Software 6.1 (Bio-Rad, Hercules, CA, USA). The intensity of immunoreactive bands was normalized via the loading control (HRP-linked β-actin (D6A8); 1:400; #12620; Cell Signaling, Danvers, MA, USA).

#### 2.5.3. LC-MS Analysis

All LC-MS analyses were collected at the Drug Discovery Platform, Research Institute of McGill University Health Centre. All reagents and solvents were LC-MS grade. OmniSolv^®^ acetonitrile (ACN) and methanol (MeOH) were sourced from MilliporeSigma (Burlington, MO, USA) and Optima™ formic acid from Fisher (Fisher Chemical™). Ultra-pure deionized water was obtained from the Millipore Milli-Q water system (MilliporeSigma, Burlington, MO, USA).

##### Standards and Solutions Preparations

Individual stock solutions of ZR2002 and Nitro-RB10 (IS) were prepared in DMSO at 10 mg/mL and, after, in MeOH at a concentration of 100 μg/mL. Intermediate dilutions were prepared using ACN:water (35:65, v:v). All stock and working solutions were stored at −20 °C and brought to room temperature before use.

##### Calibration Standards and Quality Control (QC) Sample Preparation

The dynamic range for the calibration curve ranged from 0.4 to 150 ng/mL, including 0.4, 1, 5, 10, 50, 80, 100 and 150 ng/mL in plasma and tumor matrix. These standards were prepared by spiking an appropriate volume of a standard spiking solution into the blank extracted plasma or tumor and vortexing. A total of 3 levels of QC samples were run during the analysis, including a low QC of 1 ng/mL, a mid QC of 10 ng/mL and a high QC of 100 ng/mL. These QC samples were prepared by spiking an appropriate volume of the spiking solution into the matrix blank. An appropriate volume of the IS spiking solution was then spiked into the calibration standards and QC samples to generate a final IS concentration of 15 ng/mL in the matrix. All samples were vortexed gently, completing the sample preparation process.

##### Plasma Sample Preparation

An amount of 50 μL of each plasma sample was deproteinized with 200 µL of cold ACN. The samples were mixed for 10 s, shaken automatically for 10 min and centrifuged for 10 min at 15,000 rpm (21,130 rcf) at 4 °C. After centrifugation, the supernatants were loaded into Captiva-Enhanced Matrix Removal (EMR)-Lipid cartridges (1.0 mL; 40 mg; Agilent, Santa Clara, CA, USA) and settled for 5 min. The supernatants were eluted by applying a positive pressure (2 psi for 2 min) using a positive pressure manifold (PPM 96 processor, Agilent Techn.). The cartridges were washed with ACN:water (8:2, v:v) 100 μL, at 2–4 psi for 2 min, followed by a 1-min period of high pressure (6–9 psi) to drain the cartridges. The elutes were transferred to new tubes and dried under vacuum using a Centrivap sample concentrator (Centrivap Complete^TM^, Labconco Corporation, Kansas City, MO, USA) at room temperature. The dried samples were stored at −20 °C before LC-MS analysis. On the day of analysis, the samples were reconstituted in 100 µL of MeOH, vortexed for 10 s, sonicated for 5 min (sonic bath, Symphony, VWR International, Radnor, PA, USA) and centrifuged for 5 min at 15,000 rpm. The supernatants were diluted 60 times in 35% ACN, and an appropriate volume of the IS spiking solution was then spiked into the samples to generate a final IS concentration of 15 ng/mL. At the end of the process, all samples were diluted 120 times. These final solutions were transferred to autosampler vials (350 µL, Thermo Fisher Scientific, Waltham, MA, USA), and 10 µL of each was injected into the LC-MS system.

##### Tumor Sample Preparation

A weighted piece of tumor was crushed in a mortar with liquid nitrogen. To the tumor powder, 150 μL of water was added before deproteinizing the mixture with 600 µL of cold ACN. The samples were mixed for 10s, sonicated with ice in a sonication bath for 30 min to 1 h (Sonicate bath, VWR, International, Radnor, PA, USA) and centrifuged for 10 min at 15,000 rpm (21,130 rcf) at 4 °C (Centrifuge 5424 R, Eppendorf). After centrifugation, the supernatants were loaded into Captiva-Enhanced Matrix Removal (EMR)-Lipid cartridges (1.0 mL; 40 mg; Agilent) and settled for 5 min. The supernatants were eluted by applying a positive pressure (2 psi for 2 min) using a positive pressure manifold (PPM 96 processor, Agilent Techn.). The cartridges were washed with ACN:water (8:2, v:v) 240 μL at 2–4 psi for 2 min, followed by a 1-min period of high pressure (6–9 psi) to drain the cartridges. The elutes were transferred to new tubes and dried under vacuum using a Centrivap sample concentrator (Centrivap Complete^TM^, Labconco, Kansas City, MO, USA) at room temperature. The dried samples were stored at –20 °C before LC-MS analysis. On the day of analysis, the samples were reconstituted in 100 µL of MeOH, vortexed for 10 s, sonicated for 5 min (sonic bath, Symphony, VWR, International, Radnor, PA, USA) and centrifuged for 5 min at 15,000 rpm. The supernatants were diluted 60 times in 35% ACN and an appropriate volume of the IS spiking solution was then spiked into the samples to generate the final IS concentration of 15 ng/mL. At the end of the process, all samples were diluted 60 times. These final solutions were transferred to autosampler vials (350 µL, Thermo Fisher Scientific, Waltham, MA, USA) and 10 µL of each was injected into the LC-MS system.

##### Metabolites Identification with LC-MS

LC-MS analyses were performed with an ESI-QTOF-MS system (Impact II, Bruker, Billerica, MA, USA) coupled with a liquid chromatography system (1290 Infinity, Agilent) and equipped with a reversed-phase InertSustain AQ-C18 column (100 × 2.1 mm; 3 µm) GL Sciences Inc. Mobile phases were water with 0.1% formic acid (A) and acetonitrile with 0.1% formic acid (B). The following gradient elution method was used: 5% B from 0 to 1 min, 5−60% B from 1 to 12 min, a 60% B plateau for 12 to 16 min, 60−80% B from 16 to 20 min, an 80% B plateau for 3 min and a return to initial conditions with equilibration for 6 min. The column temperature was set at 30 °C, and the flow rate was 0.4 mL/min. The samples were kept in an autosampler at 4 °C. Each sample was injected at a volume of 10 μL. The operating parameters of the mass spectrometer were as follows: positive spray voltage at 4000 V, dry temperature at 250 °C, dry gas flow at 10 L/min and nebulizer at 3 bar. The data were collected with a mass range from 50 to 1000 *m*/*z*. The instrument was calibrated using a 10 mM sodium formate solution before the runs, and, at the beginning of each run, this solution was injected as an internal calibrant for MS spectrum calibration. The LC-MS acquired data were processed using DataAnalysis Version 5.2 software (Bruker) to extract the mass spectra from the sample raw data.

##### Quantification by LC-MS/MS

The LC-MS/MS system consisted of a triple quadrupole MS system (EVOQ Elite, Bruker, Billerica, MA, USA) coupled with an ultrahigh-performance liquid chromatography pump (Advance, Bruker) and equipped with a reversed-phase chromatography column. Chromatography was performed with an InertSustain AQ-C18 column (100 × 2.1 mm; 3 µm-GL; Sciences Inc., Shinjuku-ku, Tokyo, Japon) at 30 °C at a flow rate of 0.4 mL/min using a gradient solvent of mobile phase A, H_2_O + 0.1% formic acid., and mobile phase B, acetonitrile + 0.1% formic acid. The LC method starts at 35% B for 0.5 min, then increases, firstly, to 50% B over 2 min and then to 85% in 1 min, followed by a plateau at 85% B for 2.5 min, and then returns to initial conditions for 4 more minutes. The total run time was 10 min, and each sample was injected in triplicate at a sample volume of 10 μL. The introduction of the matrix components in the MS unit was reduced by the use of a diverter valve, and the eluent flow was sent to waste during the first 0.5 min and after 5.14 min of the run. The temperature of the autosampler was thermostated at 10 °C. The injection needle was washed for 20s (×2) with methanol:water (7:3) and for 20s (×2) with a mixture of 4 solvents [methanol:acetonitrile:water:isopropanol (1:1:1:1)] after each injection. Two sets of calibration standards were run at the beginning and the end of the sequence, bracketing the three levels of QC samples from a low to a high level. Blank matrix samples were run for carryover assessment. The operating parameters of the mass spectrometer were as follows: ESI source in positive mode, positive spray voltage at 4500 V, cone temperature at 350 °C, cone gas flow at 20 psi, heated probe temperature at 400 °C, probe gas flow at 40 psi and nebulizer gas flow at 60 psi. The mass spectrometer was used in the multiple reaction monitoring mode (MRM). For ZR2002, two transitions were followed: 377.1→340.9 (CE 29 eV–quantifier ion) and 377.1→260.9 (CE 39 eV–qualifier ion). Additionally, for Nitro-RB10, two transitions were followed: 344.9→298.8 (CE 26 eV–quantifier ion) and 344.9→218.9 (CE 44 eV–qualifier ion).

Experimental data processing and analysis were performed using MSWS software (Bruker Daltonics, version 8.2.1) and Microsoft Excel (Microsoft Inc., Redmond, WA, USA version 2013). Quantification of ZR2002, based on peak areas, was performed following an internal calibration curve. A total of 8 points of calibration were used to produce a standard curve, and a cubic curve fit was demonstrated in the dynamic range of 0.4 to 150 ng/mL in plasma and tumor matrix with a weight of 1/x. The curve showed excellent fitting over the calibration range, with an R^2^ of >0.99.

### 2.6. Statistical Analysis

Statistical analysis was performed using GraphPad Prism (GraphPad Software 9.0.1, San Diego, CA, USA). The data are expressed as mean ± SEM. The in vitro (SRB) data are representative of three independent experiments run in triplicate. IC50s were calculated using a nonlinear regression model with a four-parameter logistic equation using GraphPad software 9.0.1. The LC-MS is representative of 4 samples. A Student’s t-test was used to compare the parameters of the different groups, and an unpaired t-test was used for continuous variables after D’Agostino and Pearson’s normality tests, or a nonparametric Mann–Whitney test if the distribution was not normal. Reported *p*-values were 2-sided and considered significant when *p* < 0.05.

## 3. Results

### 3.1. Growth Inhibition

**Figure A1:**
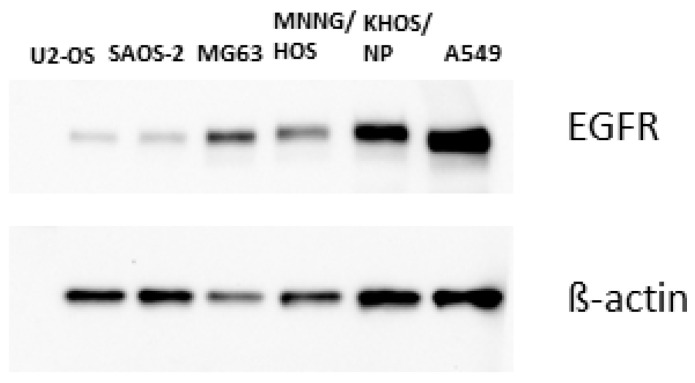
Western blot of EGFR expression in osteosarcoma cell lines. β-actin was used as internal control, and the overexpression of EGFR in the lung cancer cell line, A549, was introduced as positive control.

Given the low-mild expression of EGFR reported in different osteosarcoma cell lines, including Saos-2, the clinical EGFR inhibitor gefitinib has been previously evaluated in vitro and found to have a moderate inhibitory effect on cell proliferation [31]. However, as osteosarcoma cells express EGFR, the combination of gefitinib with chemotherapy has been reported to increase antitumor cell growth in vitro [32]. Likewise, herein, we sought to determine the growth inhibitory profiles in a panel of osteosarcoma cells with moderate to high EGFR expression, including the U2-OS, MG-63, MNNG/HOS and KHOS/NP osteosarcoma cell lines (Figure A1). In vitro, the mustard-linked combi-molecule JS61 showed an IC50 of 1.39–12.53 μM and the di-substituted JS84 of 0.18–5.04 μM in a growth inhibition assay (Table 1). By contrast, the IC50 of ZR2002 remained in the submicromolar range across the panel of different cell lines (0.08–0.45 uM). Within this range of IC50 values, ZR2002 appeared to be 3–14- and 17–36-fold more potent than JS84 and JS61, respectively. However, based on the metabolic pathways depicted in Figure 1 that outline their potential conversions into ZR2002, we expected them to be stable carriers of ZR2002 to tumor cells in vivo following metabolic dealkylation. It should be noted that ZR2002 was consistently more potent than the clinical EGFR inhibitor gefitinib, the nitrogen mustard chlorambucil, and their corresponding combination.

### 3.2. In Vivo Efficacy

**Figure 2:**
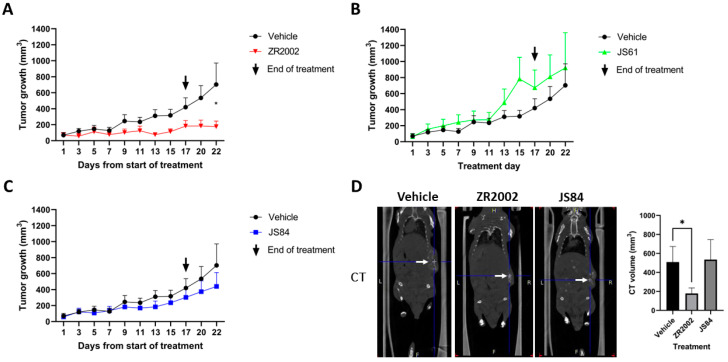
Antitumor activities of ZR2002, JS61 and JS84. Quantification of tumor growth measured with caliper in (**A**) ZR2002-treated group, (**B**) JS61-treated group and (**C**) JS84-treated group compared with vehicle group in each graph. (**D**) Representative coronary sections of CT images for ZR2002, JS84 or vehicle-treated mice (white arrows indicate the tumor) with CT tumor volume quantification on day 22. Data are expressed as means ± SEM. * *p* < 0.05 between the two groups.

**Figure A2:**
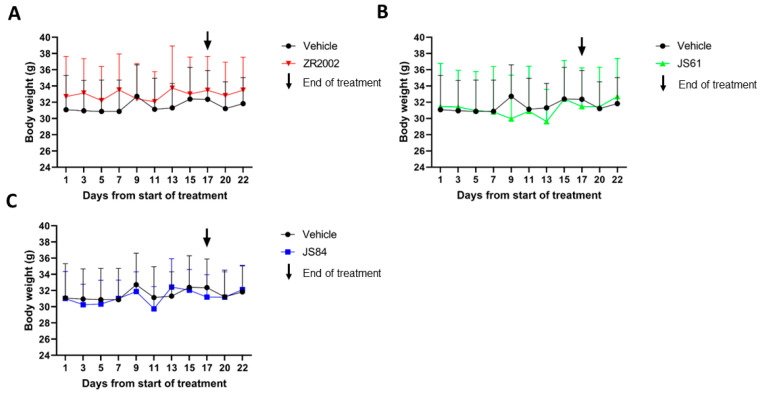
Body weights of mice treated (N = 7) with the combi-molecules. (**A**) ZR2002 versus vehicle. (**B**) JS61 versus vehicle. (**C**) JS84 versus vehicle. Data are expressed as means ± SEM.

Since we aimed to explore if our 6-N,N-di-substituted combi-molecules were transformed into mono-substituted combi-molecules in vivo, which could enhance their potency, we administered the three combi-molecules individually to mice (ZR2002, JS61 and JS84) on an equidose basis and compared their growth inhibitory activities. All 3 drugs were well tolerated with no significant weight loss throughout treatment at a dose of 80 mg/kg (Figure A2). This dose was approximately twice below the maximum tolerated doses of ZR2002, JS61 and JS84 that were previously evaluated [16]. At the end of treatment with ZR2002, tumor size was significantly reduced (*p* < 0.05), indicating strong antitumor activity for ZR2002. By contrast, no significant growth inhibition was seen in animals treated with JS61 and JS84 (*p* > 0.05), as measured with a caliper (Figure 2A–C) and CT scan (Figure 2D).

**Figure 3:**
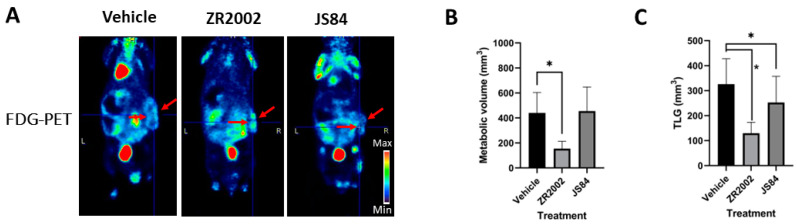
Tumor volume measurement with FDG-PET on day 22. FDG-PET scan was performed one day prior to the sacrifice of vehicle-, ZR2002- and JS84-treated groups. (**A**) Representative coronary sections of FDG-PET images for ZR2002-, JS84- or vehicle-treated mice (red arrows indicate the tumor) on day 22. (**B**) Metabolic volume. (**C**) Total lesion glycolysis (TLG). Data are expressed as means ± SEM. * *p* < 0.05 between the two groups.

The Saos-2 tumor burden appeared to be extended; therefore, we surmised that some tumors were growing internally. Thus, we performed CT/PET imaging in order to fully visualize and accurately measure the sizes of the tumors [33]. Our analysis focused on JS84, which showed minor antitumor activity with a caliper, and ZR2002, which induced significant tumor reduction (*p* < 0.05) on day 22 (Figure 2C). As observed with the caliper measurement, the results showed that ZR2002 led to a significant reduction (*p* < 0.05) in CT and metabolic volume (MV) (Figure 2D and Figure 3B). By contrast, the tumor volumes treated with JS84 were not statistically different in comparison with the vehicle. The global tumor glycolysis was calculated as total lesion glycolysis (TLG), the product of the MV and the SUV mean. ZR2002- and JS84-treated tumors showed a reduction in TLG, indicating that these two combi-molecules reduced the FDG uptake in the whole tumor volume.

### 3.3. Pharmacodynamics

**Figure 4:**
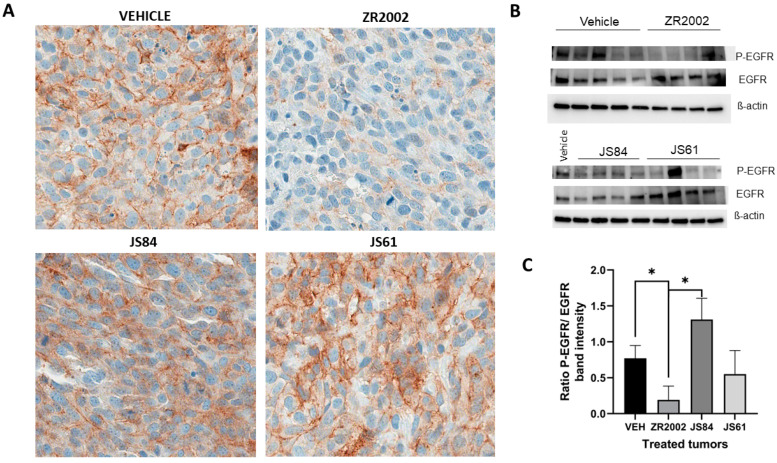
Immunohistochemistry and Western blot analysis of the effects of ZR2002, JS84 and JS61 on tumor tissues. ZR2002 decreases the phosphorylation of EGFR (P-EGFR). (**A**) Representative sections of Saos-2 tumors stained for P-EGFR. (**B**) Western blots for P-EGFR (Tyr 1068) and EGFR, where β-actin was used as internal control. Each band corresponds to different tumor lysates. (**C**) Quantification of Western blot: ratio of band intensity between P-EGFR and EGFR total protein. Data are expressed as means ± SEM. * *p* < 0.05 between the two groups.

**Figure 5:**
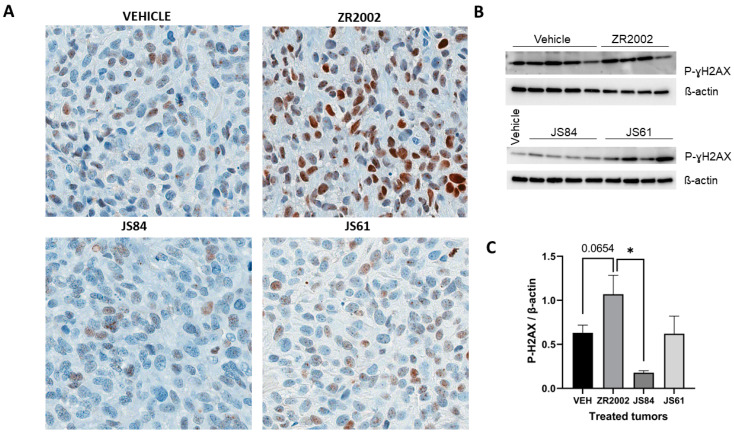
Immunohistochemistry and Western blot analysis of the effects of ZR2002, JS84 and JS61 on tumor tissues. ZR2002 increases the phosphorylation of ɣ-H2AX (P-ɣH2AX). (**A**) Representative tumor sections of Saos-2 tumors stained for P-ɣH2AX. (**B**) Western blots for P-ɣH2AX and β-actin, which was used as internal control. (**C**) Quantification of Western blots: ratio of band intensity between P-ɣH2AX and β-actin. Data are expressed as means ± SEM. * *p* < 0.05 between the two groups.

In order to elucidate the molecular mechanism of action of ZR2002 in the tumor, we analyzed the effects of its two main components, i.e., the 2-chloroethyl DNA-damaging moiety with P-H2AX and the quinazoline-based scaffold as an EGFR inhibitor. These effects were determined using immunochemistry and Western blotting in tumor samples obtained from animals treated with ZR2002, JS61, JS84 or the vehicle (N = 4) 3 h prior to sacrifice. The EGFR phosphorylation of tyrosine (Tyr) 1068 was reduced in the ZR2002-treated tumors in comparison with the vehicle group (*p* < 0.05 via Western blot analysis) (Figure 4), and ɣ-H2AX phosphorylation was enhanced in comparison with the control, even if it was not statistically significant (*p* > 0.05) (Figure 5). By contrast, tumors from animals treated with JS61 and JS84 showed detectable EGFR phosphorylation of Tyr 1068 and low levels of H2AX phosphorylation.

### 3.4. Pharmacokinetics: Plasma and Tumor Metabolism

**Figure 6:**
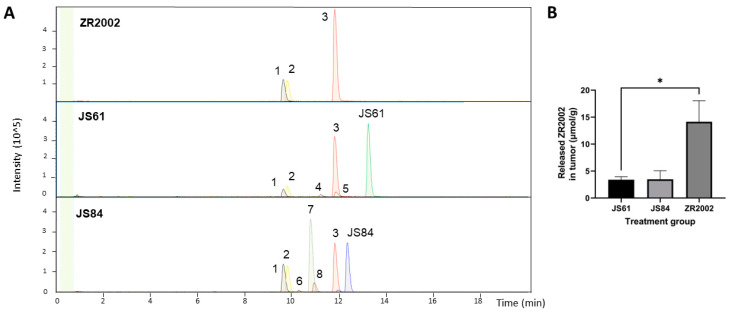
Main metabolites released in the tumors after 3 h from combi-molecule administration. (**A**) LC-MS chromatogram of the metabolization of ZR2002 (first row), JS61 (middle row) and JS84 (bottom row). Metabolites: (1) RB10, (2) RB10-N-Acetyl, (3) ZR2002, (4) JS61-OH-Cl, (5) JS61-OH-2Cl, (6) RB10-N-Acetyl-Methyl, (7) RB10-Methyl and (8) JS84-OH-Cl. The peak of JS61 intact drug is in green, while JS84’s is in blue, and the peak of ZR2002 is in red. (**B**) Quantification of ZR2002 released in the treated tumors (N = 4). Data are expressed as means ± SEM. * *p* < 0.05 between the two groups.

**Figure 7:**
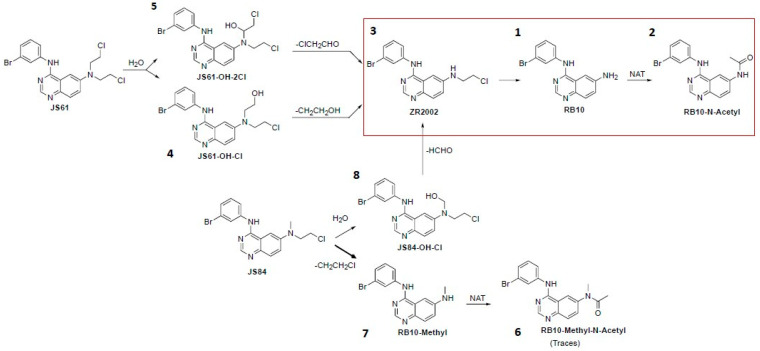
Scheme of combi-molecule metabolizations. All combi-molecules share the metabolic pathway of ZR2002, marked with the red rectangle. JS61 partially hydrolyzes, generating metabolites 4 and 5, of which concentrations are not sufficient to generate high concentrations of ZR2002 metabolite. JS84 generates high concentrations of RB10-Methyl and is not hydrolyzed enough in JS84-OH-Cl to generate high levels of ZR2002. Each metabolite has a name and number that refers to the chromatogram in Figure 6A.

**Figure A3:**
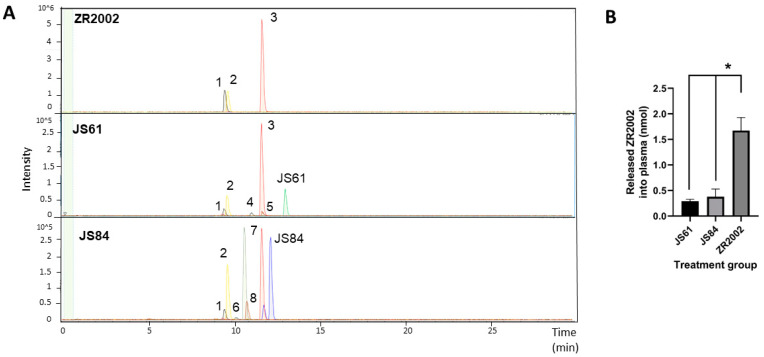
Main metabolites released in the plasma after 3 h from combi-molecule administration. (**A**) LC-MS chromatogram of the metabolization of ZR2002 (first row), JS61 (middle row) and JS84 (bottom row). Metabolites: (1) RB10, (2) RB10-N-Acetyl, (3) ZR2002, (4) JS61-OH-Cl, (5) JS61-OH-2Cl, (6) RB10-N-Acetyl-Methyl, (7) RB10-Methyl and (8) JS84-OH-Cl. The peak of JS61 intact drug is in green, while JS84’s is in blue, and ZR2002’s is in red. (**B**) Quantification of ZR2002 released in the treated tumors. Data are expressed as means ± SEM. * *p* < 0.05 between the two groups.

The plasma and intratumoral levels of each drug were determined using LC-MS/MS technology. The results show that ZR2002 is partially metabolized into its deschloroethyl metabolite “RB10” (peak 1) and RB10-N-acetyl (peak 2), as expected from previous studies [34] (Figure 6A). It should be noted that a large fraction of ZR2002 was retained in both tumors (Figure 6B) and plasma (Figure A3). JS61 also remained largely intact; however, it generated ZR2002 (peak 3) and minor metabolites, such as RB10 (1) and RB10-N-Acetyl (2), and chlorinated metabolites, such as JS61-OH-Cl (peak 4) and JS61-OH-2Cl (peak 5) (the metabolite structures are shown in Figure 7). Surprisingly, JS84 was partially intact and largely converted into RB10-Methyl (peak 7), RB10 (1) and RB10-N-Acetyl (2), with some traces of hydroxymethyl (peak 8) and N-methyl-N-acetyl metabolites (RB10-N-Acetyl-Methyl, peak 6) (Figure 6). Nevertheless, both combi-molecules JS84 and JS61 released ZR2002 (3). The concentrations of released ZR2002 in the JS61- and JS84-treated tumors were five times lower than that in the ZR2002-treated tumors (Figure 6B).

## 4. Discussion

Previous work on the dual mechanism of action of ZR2002 demonstrated that it could significantly block EGFR phosphorylation and damage DNA in a dose-dependent manner [35]. It was also shown that ZR2002 is an irreversible inhibitor of EGFR, which downregulates the MAPK and PIK3 pathways leading to strong levels of apoptosis in both breast [15] and glioblastoma cells [18]. Sharifi et al. [18] demonstrated the in vivo activity of ZR2002 at high doses (150 mg/kg) in a temozolomide-resistant glioma stem cell model. Although activity at high doses was evidence of good tolerance in vivo, it was believed that its strong potency in vitro (in the submicromolar range), could translate into efficacy at lower doses than 150 mg/kg. The significant level of deschloroethylation of ZR2002 through liver metabolism was put forth to explain the requirement for high doses to increase its dual activity. Herein, we studied molecules (JS84 and JS61) that could be prodrugs capable of generating ZR2002 through liver metabolism, thereby increasing its plasma concentration in vivo. We chose to study two prodrugs of ZR2002, either carrying an extra chloroethyl group (JS61) or a methyl group (JS84), at lower concentrations (80 mg/kg) in a human osteosarcoma mouse xenograft model. Although previous studies showed moderate EGFR inhibitory activities for JS84 and JS61 [16], we thought that their in vivo metabolism would convert them into ZR2002, thereby enhancing, overall, both the EGFR inhibitory activity and DNA alkylation.

The activity of ZR2002 was superior in comparison with all the other molecules in all cell lines, regardless of their EGFR status (Table 1). Likewise, the potency of gefitinib in our experiments did not correlate with EGFR level expression (Figure A1). This is in agreement with recent studies by Sevelda et al. [32], who observed that the blockade of EGFR by gefitinib inhibited osteosarcoma cell proliferation independently from their EGFR expression levels in osteosarcoma cell lines. Furthermore, Lee et al. [31] found that gefitinib at concentrations of up to 20 μM did not reduce the viability of several other osteosarcoma cell lines, confirming their robust resistance to gefitinib. Recently, Marshall et al. [36], in order to value EGFR expression as a target in osteosarcoma, designed a complex nanoparticle that was able to deliver doxorubicin (a DNA-damaging agent) using a conjugated EGFR antibody. ZR2002 without encapsulation targets EGFR, damages DNA and exhibits superior cytotoxicity when compared with all the drugs in all cell lines. Thus, our study reveals the discovery of a simple dual-targeting strategy that yields a molecule, ZR2002, with unique potency against osteosarcoma cells expressing various levels of EGFR.

In the Saos-2 cell line that we chose for in vivo studies, without metabolic activation, ZR2002 appears to be 7-fold and 14-fold more potent than JS84 and JS61, respectively (Table 1). This result can be explained by the fact that in the absence of metabolic enzymes, JS84 and JS61 remain intact, being able to exert the dual function (the inhibition of EGFR and DNA damage) less efficiently than ZR2002. In fact, the EGFR inhibitory potencies of JS84 and JS61 are less than that of ZR2002, and this is believed to be due to the bulkiness imposed by the methyl and chloroethyl groups of JS84 and JS61, respectively [16]. It is also known that substitutions at the N-6 position tilt the torsion angle of the substituents and the proton of the quinazoline ring, thereby decreasing the binding affinities of JS84 and JS61 to the ATP site. In addition, it was previously found that JS61 is able to induce more damage than JS84 and ZR2002, carrying a double chloroethyl moiety, even though it is believed that the EGFR-inhibitory function plays a major role in the overall potency [16]. Thus, we expected that JS84 and JS61 would act as prodrugs in vivo through metabolic dealkylation to generate and release free ZR2002 in the tumors.

When we tested the three combi-molecules in vivo, we immediately observed that the two prodrugs were not slowing down tumor growth as strongly as ZR2002 during treatment (Figure 2). Surprisingly, only ZR2002 showed a superior efficacy in inhibiting tumor growth even after 5 days after treatment when we performed CT/PET imaging. The CT and PET volume (MV) confirmed that the ZR2002-treated tumors had a reduced volume in comparison with the vehicle- or JS84-treated tumors. The FDG-PET imaging reveals that both ZR2002 and JS84 were able to statistically reduce the total glycolytic activity (TLG parameter, Figure 3C), and we speculate that the inhibition of the EGFR signaling pathway may have interfered indirectly with the reduction in the glycolytic pathway, decelerating the tumor proliferation. In fact, it has been previously observed that EGF signaling activates the first step in glycolysis with hexokinase 2 (HK2) but impedes the last step with pyruvate kinase muscle isozyme M2 (PKM2) in triple breast cancer cells [37], and that PKM2 expression is aberrantly high in osteosarcoma tissues [38]. A direct link between PKM2 expression and EGFR activity was also established in human glioblastoma specimens [39].

In order to explain the overall results, we performed LC-MS analysis to identify the drug metabolism of the three combi-molecules (Figure 6 and Figure A3). Following the administration of ZR2002, the intact combi-molecule and its anti-EGFR metabolites “RB10” and “RB10-N-Acetyl” were observed, indicating that, indeed, ZR2002 is partially deschloroethylated in vivo, losing its DNA-damaging properties. Importantly, following the administration of JS84, the formation of ZR2002 and its anti-EGFR metabolites, “RB10”, “RB10-N-Acetyl” and “RB10-Methyl”, were observed, suggesting that JS84 is also significantly dealkylated in vivo. These metabolites could not have sustained the EGFR inhibition after a few days at the end of the treatment with JS84, as confirmed by the IHC or Western blot analysis (Figure 4). Furthermore, the concentrations of the ZR2002 metabolite in the JS84-treated tumors and plasma were significantly lower than those in the ones treated with ZR2002. In conclusion, the loss of the alkylating group, which is responsible for DNA damage, and the decrease in the EGFR inhibitory potency through the massive formation of the RB10 metabolites can explain the decreased efficacy observed during the JS84 treatment (Figure 2). Likewise, following the administration of JS61, the formation of “RB10” and “RB10-N-Acetyl” reflects the extent of the deschloroethylation of ZR2002; however, the levels of these metabolites were significantly lower than those for JS84 (Figure 6). The loss of chlorine in one of the chloroethyl groups of JS61 allows us to propose that a hydroxy metabolite is perhaps associated with the loss of its alkylating (DNA-damaging) potential. Although levels of ZR2002 were detected, JS61 remained largely unmetabolized, leading to non-significant in vivo activity in the JS61-treated tumors.

The types of metabolites observed in vivo for the three combi-molecules suggest the metabolic pathways depicted in Figure 7. This allows us to conclude that the concentration of ZR2002 is maximal when it is administered structurally unmodified with an extent of deschloroethylation that is statistically less than that of JS84. As for JS61, although the extent of deschloroethylation was similar to that of intact ZR2002, JS61 was largely unmetabolized, yielding a small fraction of ZR2002 in the tumor.

## 5. Conclusions

In summary, the weak activity observed for the prodrugs in tumor growth inhibition in vivo appears to be in agreement with the extent of the drug metabolism observed for the two putative prodrug candidates JS84 and JS61. This is believed to be due to the low titer of ZR2002 delivered by the latter molecules. Herein, it is important to highlight the potency of intact ZR2002 in this aggressive osteosarcoma model, with evidence of intratumoral inhibition of EGFR and apparent DNA damage, as detected via H2AX. This indicates that critical plasma and tumor levels of ZR2002 elicit targeted modulation in tumor cells in vivo. This was seen in an osteosarcoma model known to be platinum-resistant [40,41,42], which points towards further studies in resistant sarcomas or other diseases in which platinum resistance is observed. This study conclusively demonstrated that strategies to enhance the potency of ZR2002 should not involve alkyl substitution at the N6 position of the quinazoline ring, and this is under investigation in our laboratory.

## Figures and Tables

**Table 1:** Growth inhibition potencies of the various combi-molecules against osteosarcoma cells.

Compound	IC50 (µM) ^a^				
	U2-OS	Saos-2	MG-63	MNNG/HOS	KHOS/NP
JS61	1.39 ± 0.02	1.59 ± 0.10	12.53 ± 0.61	5.86 ± 0.15	6.83 ± 0.50
JS84	0.18 ± 0.03	0.25 ± 0.04	5.04 ± 0.98	0.88 ± 0.43	1.38 ± 0.30
ZR2002	0.08 ± 0.01	0.08 ± 0.01	0.45 ± 0.08	0.31 ± 0.09	0.19 ± 0.03
Gefitinib	22.16 ± 4.42	20.62 ± 4.20	13.40 ± 0.08	10.64 ± 0.56	13.41 ± 0.45
Chlorambucil	9.59 ± 0.55	7.53 ± 0.75	13.05 ± 1.28	12.10 ± 1.01	9.25 ± 1.35
Gefitinib + chlorambucil ^b^	3.97 ± 0.84	1.96 ± 0.28	13.38 ± 4.68	10.29 ± 3.21	6.60 ± 0.19

^a^ Values represent means and SEM from three independent experiments. ^b^ Equimolar combination of chlorambucil and gefitinib.

## Data Availability

Not applicable.

## Appendix A

The appendix is an optional section that can contain details and data supplemental to the main text.
